# Pyramidal tract and alternate motor fibers complementarily mediate motor compensation in patients after hemispherotomy

**DOI:** 10.1038/s41598-020-57504-x

**Published:** 2020-01-23

**Authors:** Jennifer Gaubatz, Leon Ernst, Conrad C. Prillwitz, Bastian David, Guido Lüchters, Johannes Schramm, Bernd Weber, Rainer Surges, Elke Hattingen, Gottfried Schlaug, Christian E. Elger, Theodor Rüber

**Affiliations:** 1 Department of Epileptology, University of Bonn Medical Center, Bonn, 53127 Germany; 2 Center for Development Research, University of Bonn, Bonn, 53113 Germany; 3 Department of Neurosurgery, University of Bonn Medical Center, Bonn, 53127 Germany; 4 Institute for Experimental Epileptology and Cognition Research, University of Bonn Medical Center, Bonn, 53127 Germany; 5 Department of Neuroradiology, Goethe University Frankfurt, Frankfurt am Main, 60528 Germany; 6 Stroke Recovery Laboratory, Beth Israel Deaconess Medical Center/Harvard Medical School, Boston, 02215 MA USA; 7 Epilepsy Center Frankfurt Rhine-Main, Department of Neurology, Goethe University Frankfurt, Frankfurt am Main, 60528 Germany; 8 Center for Personalized Translational Epilepsy Research (CePTER), Goethe-University Frankfurt, Frankfurt am Main, 60528 Germany

**Keywords:** Neural circuits, Neonatal brain damage, Epilepsy

## Abstract

Motor function after hemispheric lesions has been associated with the structural integrity of either the pyramidal tract (PT) or alternate motor fibers (aMF). In this study, we aimed to differentially characterize the roles of PT and aMF in motor compensation by relating diffusion-tensor-imaging-derived parameters of white matter microstructure to measures of proximal and distal motor function in patients after hemispherotomy. Twenty-five patients (13 women; mean age: 21.1 years) after hemispherotomy (at mean age: 12.4 years) underwent Diffusion Tensor Imaging and evaluation of motor function using the Fugl-Meyer Assessment and the index finger tapping test. Regression analyses revealed that fractional anisotropy of the PT explained (*p* = 0.050) distal motor function including finger tapping rate (*p* = 0.027), whereas fractional anisotropy of aMF originating in the contralesional cortex and crossing to the ipsilesional hemisphere in the pons explained proximal motor function (*p* = 0.001). Age at surgery was found to be the only clinical variable to explain motor function (*p* < 0.001). Our results are indicative of complementary roles of the PT and of aMF in motor compensation of hemispherotomy mediating distal and proximal motor compensation of the upper limb, respectively.

## Introduction

Neuroimaging has substantially advanced our understanding of the neuronal mechanisms of functional motor recovery after brain lesions, thereby, enhancing prediction of motor recovery^1^. Diffusion Tensor Imaging (DTI) and tractography have proven useful for the *in vivo* delineation and assessment of white matter pathways^2^. Different DTI-studies have associated motor function after hemispheric lesions with the microstructural integrity of the pyramidal tract (PT) or alternate motor fibers (aMF)^3–6^. aMF are believed to constitute the imaging correlate of cortico-rubro-spinal or of cortico-reticulo-spinal pathways. They may be reconstructed by means of tractography as they descend from the precentral gyrus through the posterior limb of the internal capsule and the tegmentum pontis^7–10^. Our understanding of aMF and their role as a compensatory corticospinal system was only recently investigated in patients and has been derived as a comparative neuroanatomical approach from numerous animal studies^11–13^. Some studies have suggested that aMF could compensate for the damaged PT^6–8,14^, while other studies concluded that the portion of intact fibers of the affected PT determines the degree of motor recovery^3,4,15^. A third possibility is that both systems contribute to motor recovery. Schulz and colleagues found no interaction between diffusivity parameters indexing the microstructural status of PT and aMF in patients after stroke, and thus concluded, that the manner in which they function is “synergistic, but independent”^7^. Our hypothesis states that PT and aMF may operate synergistically by mediating distal and proximal motor functions in the lesioned brain, respectively. Dexterity, the ability to fractionate movement of individual fingers, is believed to be enabled solely by monosynaptic connections of the PT^16,17^. The polysynaptic cortico-rubro-spinal and cortico-reticulo-spinal pathways have been associated with synergistic and proximal movements^11,18,19^. We used DTI and tractography for the reconstruction and microstructural assessment of PT and bilateral aMF (crossing and unilateral/uncrossed aMF) in patients after hemispherotomy and matched healthy controls. Hemispherotomy is a hemispheric disconnection procedure, indicated as a neurosurgical *ultima ratio* option in the treatment of certain patients with therapy-refractory epilepsy^20^. Hemispherotomy patients represent an especially interesting case for the investigation of motor recovery: Their lesion occurs very early in the course of development, when the brain is thought to be most plastic, and ipsilesional influences on post-operative motor recovery may be excluded. With this study, we aim to characterize the roles of PT, unilateral/uncrossed aMF (^II^aMF; originating in the contralesional hemisphere) and crossing aMF (^x^aMF; originating in the contralesional hemisphere and crossing in the pons) in facilitating motor recovery by relating measures of motor function to tract-specific diffusivity parameters, namely fractional anisotropy (FA), and to clinical data of patients after hemispherotomy.

## Results

### Clinical explanatory variables of motor function

**Figure 1:**
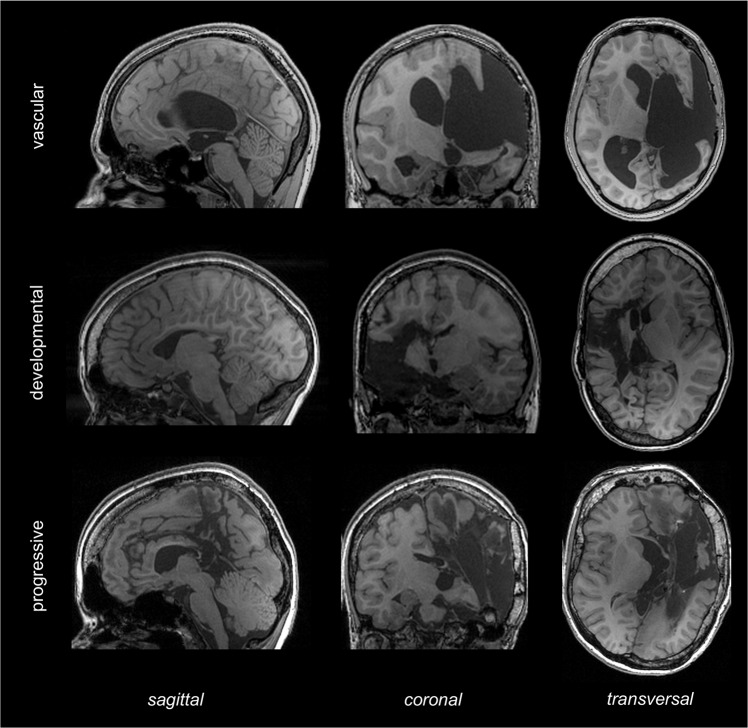
T1-weighted sequence of three exemplary patients after hemispherotomy.

Twenty-five patients who underwent a hemispherotomy using a transsylvian approach^21^ were included in this study. Patients presented with different etiologies: twelve patients suffered from perinatal strokes or perinatal intracranial hemorrhages resulting in porencephaly; six patients showed neurodevelopmental disorders such as hemimegalencephaly, Sturge-Weber syndrome or polymicrogyria; and seven patients were diagnosed with Rasmussen- or other encephalitides between the ages of 3 and 7. Anatomical T1-weighted sequence of three exemplary patients are presented in Fig. 1. Patients with development disorders underwent surgery earliest (at 39.5 months on average), whereas patients with progressive disorders underwent surgery latest (at 212.86 months on average). The characteristics of the patients are provided in Table 1.

**Table 1:** Overview: subjects.

Patients n = 25													
Etiology	ID	Lesion Side	Gender	Etiology	Age at surgery (months)	Age at scan (years)	FA			Motor Test Scores			
							PT	^II^aMF	^x^aMF	^*ue*^*MF*	^*prox*^*MF*	^*dist*^*MF*	*FTR*
vascular	1	L	M	porecenphaly	130	16	0.70	0.61	0.58	36	12	2	53.5
	2	R	M		199	19	0.61	0.51	0.47	19	6	2	32.5
	3	R	M		183	17	0.67	0.47	0.51	29	8	4	48.5
	4	L	F		162	21	0.57	0.46	0.48	28	10	2	59
	5	L	F		223	20	0.59	0.45	0.54	30	13	2	40
	6	L	F		118	20	0.69	0.57	0.55	36	10	4	60.5
	7	L	F		142	22	0.61	0.59	0.56	29	10	5	55.5
	8	R	F		131	21	0.58	0.47	0.47	26	9	2	56.5
	9	L	M		126	18	0.69	0.60	0.57	39	10	4	/
	10	R	F		303	36	0.70	0.67	0.51	17	6	0	32.5
	11	R	F		194	27	0.69	0.52	0.45	18	5	2	51
	12	L	F		110	19	0.66	0.50	0.54	31	10	3	58
	**mean ± SD**	**7 left**	**8 females**		**168.42 ± 55.81**	**21.33 ± 5.40**	**0.65 ± 0.05**	**0.53 ± 0.07**	**0.52 ± 0.04**	**28.17 ± 7.20**	**9.08 ± 2.43**	**2.67 ± 1.37**	**49.77 ± 10.27**
developmental	13	L	F	HMEG	72	25	0.62	0.46	0.48	40	11	3	55.5
	14	L	F	HMEG	4	18	0.59	0.50	0.49	29	7	3	51
	15	R	F	HMEG	9	11	0.71	0.52	0.48	50	10	6	62
	16	R	M	PLMI	125	23	0.67	0.52	0.50	22	8	2	54
	17	R	F	HMEG	8	16	0.62	0.55	0.49	28	13	2	40.5
	18	R	M	SWS	19	20	0.68	0.59	0.58	42	11	4	50
	**mean ± SD**	**2 left**	**4 females**		**39.5 ± 48.93**	**18.83 ± 5.04**	**0.65 ± 0.05**	**0.52 ± 0.04**	**0.51 ± 0.04**	**35.17 ± 10.52**	**10 ± 2.19**	**3.33 ± 1.51**	**52.17 ± 7.12**
progressive disorders	19	L	M	encephalitis	228	20	0.64	0.58	0.57	24	8	2	51.5
	20	L	F		221	20	0.56	0.48	0.48	19	8	1	39.5
	21	R	F		370	46	0.62	0.58	0.52	20	6	3	44.5
	22	L	F		89	11	0.61	0.51	0.50	21	10	3	23
	23	L	M		150	18	0.66	0.48	0.47	24	5	5	60
	24	R	F		83	12	0.67	0.50	0.52	51	15	4	58.5
	25	R	M		349	32	0.68	0.57	0.52	18	5	2	66
	**mean ± SD**	**4 left**	**4 females**		**212.86 ± 115.24**	**22.71 ± 12.37**	**0.63 ± 0.04**	**0.53 ± 0.05**	**0.51 ± 0.03**	**25.29 ± 11.57**	**8.14 ± 3.53**	**2.86 ± 1.35**	**49 ± 14.68**
total	mean ± SD	13 left	16 females		149.92 ± 98.09	21.12 ± 7.68	0.64 ± 0.05	0.53 ± 0.06	0.51 ± 0.04	29.04 ± 9.70	9.04 ± 2.70	2.88 ± 1.36	48.14 ± 14.51
Controls n = 25													
total	mean ± SD		16 females			23.31 7.28	0.59 0.04	0.44 0.03	0.45 0.04				

Abbreviations: ^dist^MF: distal motor function of the affected upper extremity; F: female; FTR: maximal finger tapping rate; HMEG: Hemimegalencephaly; L: left; M: male; PLMI: Polymicrogyria; ^prox^MF: proximal motor function of the affected upper extremity; R: right; SD: standard deviation; SWS: Sturge-Weber syndrome; ^ue^MF: motor function of the affected upper extremity; /: missing data.

**Figure 2:**
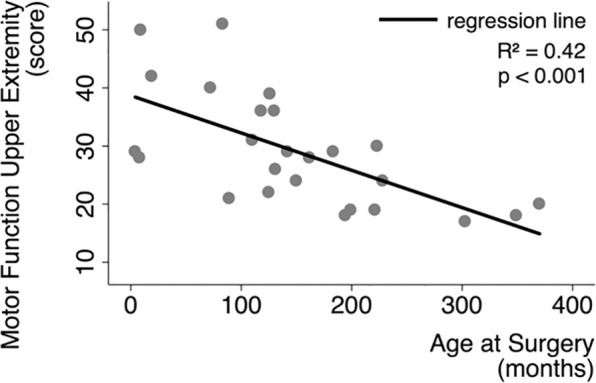
Motor function and *age at surgery*. Regressing motor function of the affected upper extremity with *age at surgery*.

A stepwise regression analysis among clinical explanatory variables revealed *age at surgery* as the only statistically significant (*p* < 0.001) explanatory variable for motor function of the upper extremity (^ue^MF). All other explanatory variables were non-significant [*affected hemisphere* (*p* = 0.848), *etiology* (*p* = 0.972), *gender* (*p* = 0.798), *time after surgery* (*p* = 0.724), *age at scan* (*p* = 0.158)], and thus removed from the model. Bootstrapping confirmed high reproducibility (*age at surgery*, *p* < 0.001) and suggested the inclusion of *age at surgery* for further regression analyses. A consecutive simple regression analysis also confirmed *age at surgery* as an explanatory variable of ^ue^MF (*p* < 0.001), with an *R*^2^ of 0.42 (see Fig. 2): the younger the subject was at surgery, the more likely it showed good motor function.

### Imaging explanatory variables of motor function

**Figure 3:**
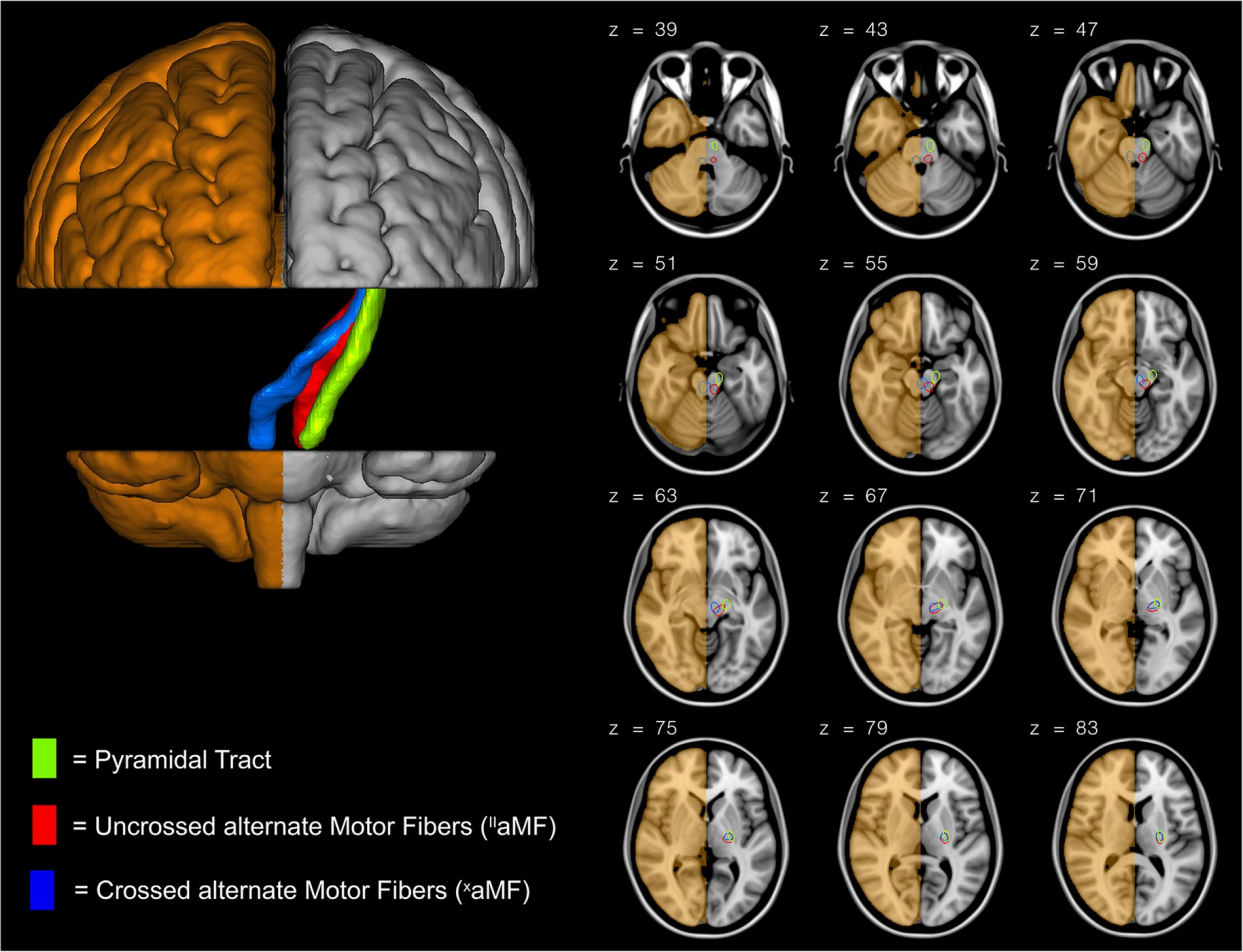
Overview of reconstructed canonical tracts. Trajectory of reconstructed canonical unilateral pyramidal tract (PT), crossed alternate motor fibers (^x^aMF) and unilateral/uncrossed alternate motor fibers (^II^aMF) as they descend from the internal capsule to the basis pontis (PT) and the tegmentum pontis (aMF). Building of canonical tracts is described in the methods section. The operated hemisphere is depicted in bronze. z indicates axial level in MNI space.

Unilateral/contralesional PT, unilateral/contralesional ^II^aMF, and ^x^aMF originating in the contralesional hemisphere and crossing the midline to the ipsilesional hemisphere in the pons could be properly reconstructed in all subjects and tract-specific FA could be extracted (see Fig. 3 for canonical tracts of patients).

**Figure 4:**
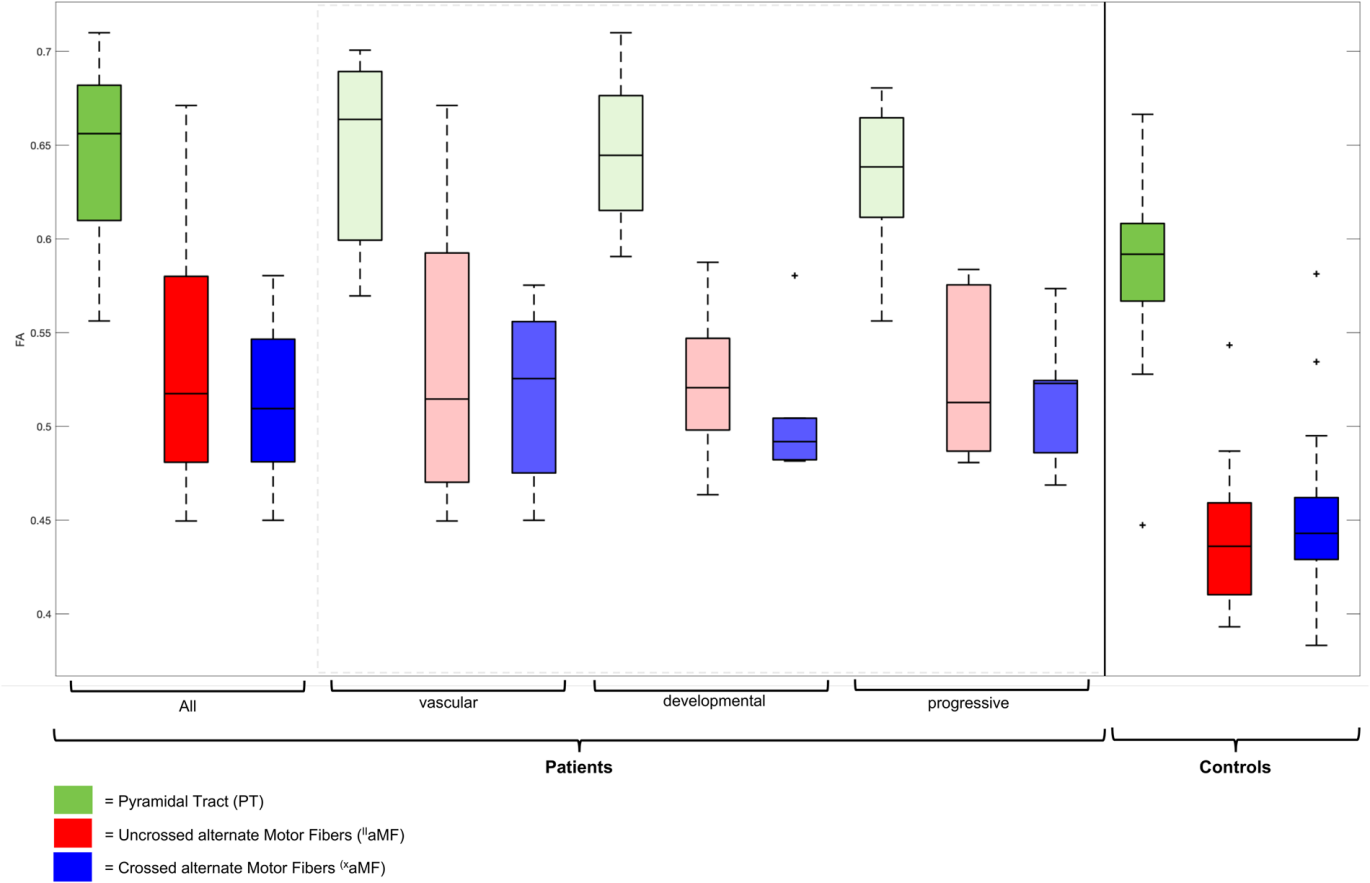
Boxplot of tract-wise FA values of patients and controls. Patients show higher FA values than controls and PT shows higher FA values than aMF-tracts (all *p* < 0.001). No statistically significant FA differences between patient subgroups *(vascular | developmental | progressive disorders)* were found (all *p* > 0.5). FA: fractional anisotropy.

Tract-wise FA showed statistically significant differences between the PT and aMF-tracts and between patients and controls: FA values of the PT were higher than FA values of aMF-tracts and FA-values of patients were higher than those of controls (all *p* < 0.001; see Fig. 4). No statistically significant FA differences between patient subgroups were found (all *p* > 0.5).

As *age at surgery* was found to be a relevant explanatory variable of motor function, it was included as an additional independent variable in all four multiple regression analyses computed to explain global (^ue^MF, first regression analysis), proximal (^prox^MF, second regression analysis), distal motor function (^dist^MF, third regression analysis) and maximal finger tapping rate (FTR as an additional measure of distal motor function, fourth regression analysis) of the upper extremity based on tract-specific FA of PT, of ^II^aMF, and of ^x^aMF. All independent variables were found to be statistically significant explanatory variables of ^ue^MF. This model with four explanatory variables accounted for 65% of the variance (R^2^) of ^ue^MF and showed positive effects of PT, ^x^aMF, and negative effects of ^II^aMF and *age at surgery*. For ^prox^MF, only ^x^aMF (*p* = 0.001) and *age at surgery* (*p* = 0.004) were significant independent explanatory variables showing positive and negative effects, respectively. For ^dist^MF, only PT (*p* = 0.050) and *age at surgery* (p = 0.049) were significant independent explanatory variables also showing positive effects and negative effects, respectively. The former model accounted for 60% of variance in ^prox^MF and the latter for 46% of variance in ^dist^MF. The only independent explanatory variable of FTR was PT (*p* = 0.027, *R*^2^ = 0.32) showing positive effects. With a variance inflation factor (VIF) < 2.5 for all variables in all regressions, we had no reason to assume multicollinearity. All results, VIF for all explanatory variables, and bivariate correlation coefficients are displayed in Table 2.

**Table 2:** Results of multiple linear regression analyses after bootstrapping, complemented by measures of multicollinearity and bivariate correlation coefficients.

Model and Data	Coefficient	SE	*Z*	*p*	+95% CI	−95% CI		R^2^	seed
^**ue**^**MF: main effects**								0.651	421670
**PT**	**77.580**	**37.512**	**2.07**	**0.039**	**4.057**	**151.102**	**0.201**		
^**X**^**aMF**	**114.941**	**38.725**	**2.97**	**0.003**	**39.040**	**190.841**	**0.283**		
^**II**^**aMF**	**−66.612**	**33.406**	**−1.99**	**0.046**	**−132.087**	**−1.137**	**0.166**		
***age at surgery***	**−0.055**	**0.014**	**−3.83**	**0.000**	**−0.083**	**−0.027**	**0.437**		
^**prox**^**MF: main effects**								0.602	905414
PT	−6.915	12.539	−0.55	0.581	−31.491	17.661	0.015		
^**X**^**aMF**	**54.181**	**16.657**	**3.25**	**0.001**	**21.534**	**86.829**	**0.402**		
^II^aMF	−18.567	15.058	−1.23	0.218	−48.080	10.947	0.106		
***age at surgery***	**−0.017**	**0.006**	**−2.89**	**0.004**	**−0.028**	**−0.005**	**0.363**		
^**dist**^**MF: main effects**								0.464	13587
**PT**	**43.873**	**22.400**	**1.96**	**0.050**	**−0.029**	**87.776**	**0.172**		
^X^aMF	36.054	28.083	1.28	0.199	−18.988	91.095	0.091		
^II^aMF	−25.565	24.176	−1.06	0.290	−72.949	21.819	0.070		
***age at surgery***	**−0.023**	**0.012**	**−1.97**	**0.049**	**−0.046**	**0.000**	**0.258**		
**FTR: main effects**								0.322	725623
**PT**	**123.875**	**55.998**	**2.21**	**0.027**	**14.121**	**233.629**	**0.204**		
^X^aMF	76.688	69.772	1.10	0.272	−60.062	213.439	0.063		
^II^aMF	−101.202	66.122	−1.53	0.126	−230.798	28.395	0.156		
*age at surgery*	−0.001	0.025	−0.04	0.967	−0.050	0.048	<0.001		
**VIF**	**variables**	**Pearson’s r (p-value)**							
1.51	**PT**	−0.028 (1)	0.554 (0.024)	0.321 (0.710)					
1.55	^**X**^**aMF**	0.021 (1)	0.581 (0.014)						
2.21	^**II**^**aMF**	0.242 (1)							
1.13	***age at surgery***								
		***age at surgery***	^**II**^**aMF**	^**X**^**aMF**	**PT**				

Bold font indicates *p* ≤ 0.05. Abbreviations: ^II^aMF: fractional anisotropy of unilateral/uncrossed alternate motor fibers; ^X^aMF: fractional anisotropy of alternate motor fibers originating in the contralesional hemisphere and crossing in the pons; CI: confidence interval; ^dist^MF: distal motor function of the affected upper extremity; FTR: maximal finger tapping rate; ^prox^MF: proximal motor function of the affected upper extremity; SE: standard error; ^ue^MF: motor function of the affected upper extremity; PT: fractional anisotropy of the pyramidal tract; VIF: variance inflation factor; η^2^_p_: partial eta squared, η^2^_p_ can be interpreted as the proportion of effect + error variance that is attributable to the effect.

## Discussion

In summary, higher FA in the PT was associated to better distal motor function and faster finger tapping; and higher FA in ^x^aMF explained better proximal motor function. In contrast, higher FA in ^II^aMF explained lower overall motor functions; and higher *age at surgery* predicted lower overall, proximal, and distal motor functions.

### The hemispherotomy case

When compared to those patients who have had a stroke and are more commonly subject to studies on motor recovery, hemispherotomy patients present two particularly interesting characteristics as study subjects: first, brain lesions leading to hemispherotomy were typically acquired pre-, perinatally or in early childhood, at a time when the motor system has not yet fully matured and the brain is thought to be most plastic. Second, the hemispherotomy itself results in the contralesional hemisphere as the only possible mediator of post-surgical motor compensation. Hemispherotomy patients are particularly intriguing because they face a maximally invasive trigger of neuroplasticity at a time when the brain is thought to be maximally plastic.

### Age at surgery

Among *time after surgery*, *age at surgery*, *gender, affected hemisphere (left | right)*, and *etiology (vascular | developmental | progressive disorders)*, only *age at surgery* could explain ^ue^MF, ^prox^MF or ^dist^MF after hemispherotomy. The non-significance of *gender* and *affected hemisphere* does not require interpretation. *Time after surgery* most likely is not significant as the mean time between *age at surgery* and *age at scan* was 8.63 years and patients are thought to have reached a static phase of recovery by then. However, the non-significance of *etiology* is somewhat unexpected, considering accruing evidence for better motor function of brain lesions occurring earlier in the life-span (i.e., in-born as compared to acquired pathologies). The reason most likely lies in the broad three-tier classification *(vascular | developmental | progressive disorders)* and the considerable variance of age of lesion within the classes. Not surprisingly, *age at surgery* differs between patient subgroups (*developmental* < *vascular* < *progressive disorders*). It is, thus, open to discussion, whether *age at surgery* represents a surrogate marker for age of lesion (as earlier lesion leads to earlier surgery) or as to what extent hemispherotomy itself is a stimulus for neuroplastic reorganization (presuming that earlier hemispherotomy occurs to a more plastic brain) in addition to the underlying lesion. Regarding the primarily inhibitory interhemispheric influences produced via transcallosal pathways, it has been suggested that the surgical deafferentation may result in a disinhibition of the contralesional hemisphere allowing it to undergo substantial neuroplastic reorganization^22^.

### Interpretation of diffusivity parameters

The interpretation of our DTI results is based on the idea that FA informs the microstructural status of white matter tracts. Diffusivity parameters have been equally used to describe white matter degeneration and to pinpoint beneficial microstructural white matter alterations, which fall in the realm of neuroplasticity. Generally, reduced diffusion anisotropy is interpreted as a sign of lesion-induced or age-related degeneration after the maturational peak^23^, whereas increased diffusion anisotropy is thought to mirror compensatory and training-induced plastic white matter alterations or white matter maturation^24,25^. In our study, patients show higher FA values as compared to controls, most likely indicating the result of preceding neuroplastic reorganization. The observed structure–function relations are indicative of the functional relevance of the diffusivity alterations seen.

### PT

FA of the PT explained ^ue^MF, ^dist^MF and FTR. FTR, as ^dist^MF, is seen as an indicator of dexterous hand function. The most likely structural correlate of reconstructed PT is the uncrossed portion of the contralesional and uncrossed PT^26^. The PT is bilaterally organized at birth, but uncrossed connections are pruned around the age of one and a half years in normal development^27^. Early lesions to the motor system lead to the preservation of these normally transient ipsilateral connections^28^. Several electrophysiological and imaging studies have implicated this pathway as mediator of motor recovery after hemispheric lesions^15,29^. The pyramidal system is believed to be an evolutionary prerequisite for dexterous hand function including grasping^16^, which is why their role as a mediator of distal arm motor function is particularly plausible.

### aMF

FA of ^x^aMF was positively associated with ^prox^MF and ^ue^MF. FA of ^II^aMF was negatively associated with ^ue^MF. The cortico-rubro-spinal and the cortico-reticulo-spinal systems have both been described as anatomical substrates of aMF. In previous DTI-studies, only ^II^aMF has been investigated and tract-specific FA was found to be inversely related to motor function^6,7^. In this study, both ^II^aMF and ^x^aMF were investigated. ^x^aMF is of special interest as evidence for post-lesional neuroplastic reorganization of crossing rubro-spinal fibers is manifold: A myriad of animal studies investigating subcortical reinnervation has examined projections originating in the primary motor cortex, which after hemidecortation, pyramidotomy or cortical infarcts can sprout fibers crossing the midline and targeting the ipsilesional (!) red nucleus^13,30,31^. As fibers from the magnocellular part of the red nucleus also cross the midline (decussation of Forel), cortico-rubro-spinal fibers from the contralesional hemisphere, sprouting to the ipsilesional red nucleus, cross twice on their trajectory from the cortex to the spine and may, thus, exert control over contralesional/paretic side. Results of previous studies and of the current study together lend support to the idea that ^x^aMF corresponds to crossing cortico-rubral (!) fibers. The crossing of rubro-spinal (!) fibers from the magnocellular part of the red nucleus, however, cannot be visualized as the caudal extension of anatomy covered by DTI-volumes is limited. A more strictly unilateral route is discussed for the bilaterally organized cortico-reticulo-spinal fibers^18,29^, hence, possibly represented by ^II^aMF. Whereas the positive association found between tract-specific FA of ^x^aMF and ^prox^MF/^ue^MF puts additional weight on our interpretation of the anatomical correlates of aMF, the inverse correlation between tract-specific FA of ^II^aMF and ^ue^MF is more difficult to conceive. Notably, this negative association has been found in earlier studies^6,7^ and has been interpreted as stronger compensatory (but lastly ineffective) effort in more severely affected patients. It should be noted that FA of ^II^aMF was only found to explain ^ue^MF (and not ^prox^MF or ^dist^MF).

### Limitations

The scope of the current study is set by the familiar limitations of DTI and of tractography^2^. Challenges lie in the verification of virtual fiber bundles as well as in the interpretation of diffusivity measures in terms of their underlying anatomical substrates. The anatomical substrates of ^x^aMF and ^II^aMF and their functional role in motor recovery in particular should be further elucidated. It should be noted, that the inference on the underlying anatomical correlate is made solely on the basis of the anatomical trajectory of the reconstructed tracts and on their functional profile as derived from the observed relationship between diffusivity parameters and motor function. A further limitation of this study is due to its cross-sectional design. This design does not allow to rule out the alternative (yet unlikely) hypothesis that structural alterations observed predate the hemispherotomy or the underlying lesion and, thus, represent a marker of successful post-operative or post-lesional rehabilitation.

### Conclusion and outlook

**Figure 5:**
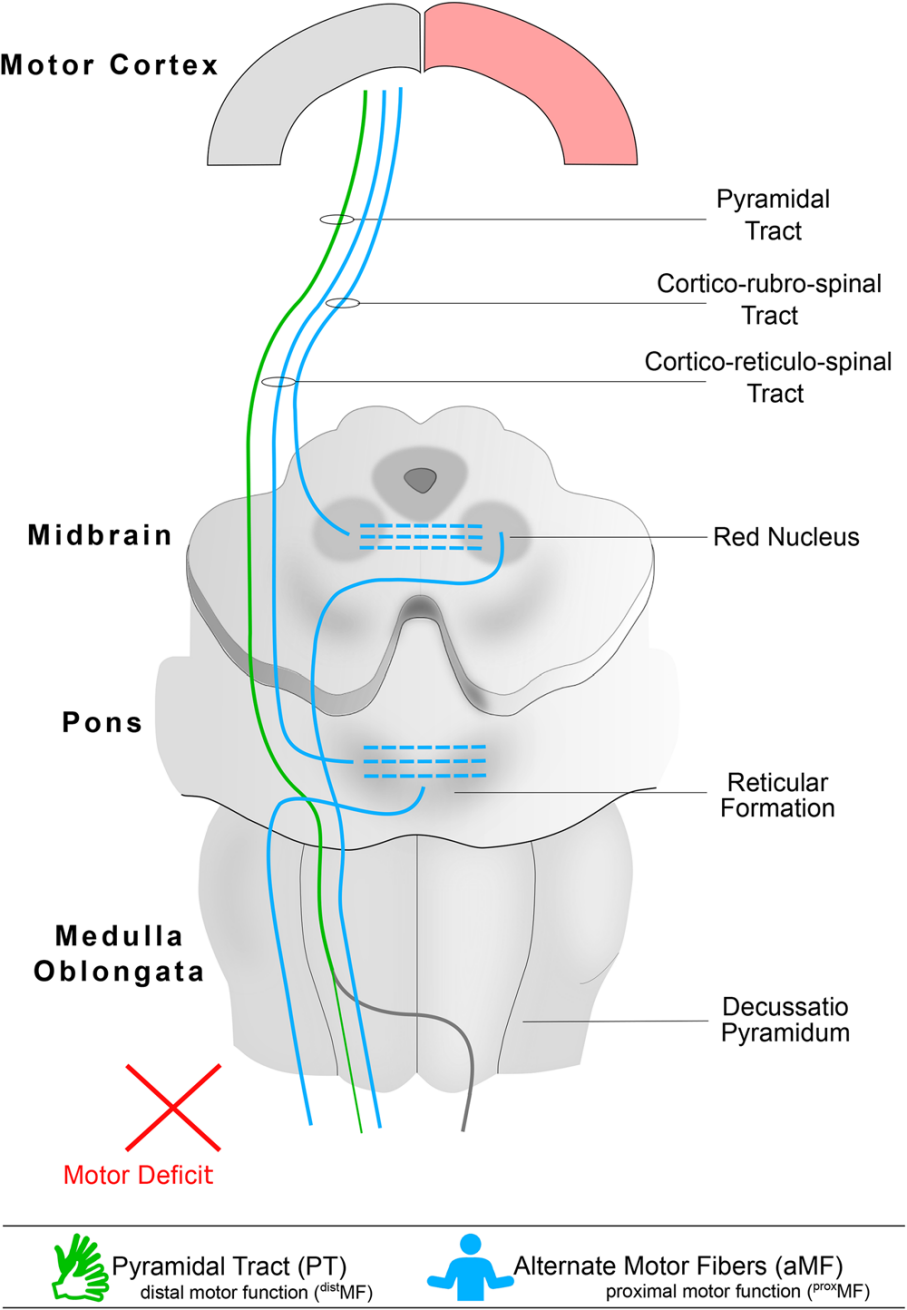
Schematic of descending pathways mediating motor recovery after hemispherotomy as suggested by this study. The operated hemisphere is marked red.

Our results suggest complementary and quite possibly synergistic roles of unilateral PT and ^x^aMF in mediating motor functions of the affected limb after large hemispheric lesions; neither unilateral PT^15^ nor aMF^6^ alone mediate motor function after hemispheric lesion. Judging by our results, motor recovery is driven by exploiting the functional diversity of the motor network with different systems mediating different functions. PT most likely corresponds to the uncrossed part of the pyramidal tract and mediates ^dist^MF. The neuroanatomical substrate of aMF most likely is cortico-rubro-spinal- and cortico-reticulo-spinal fibers mediating ^prox^MF. One should be wary of inferences on neuroanatomical underpinnings of ^x^aMF and ^II^aMF, however, based on previous animal and patient studies, it is suggested that ^x^aMF corresponds to crossing cortico-rubral fibers. Our interpretation of the results is schematically summarized in Fig. 5.

Investigating motor function after hemispherotomy may result in predictive models helpful in the preoperative evaluation of post-surgery motor outcome. More importantly, our nascent understanding of motor recovery may inspire the development of rehabilitative therapies. Williams and colleagues have speculated that the “cocorticospinal phase of the red nucleus/the rubrospinal system […] may provide a protracted window opportunity to intervene with the therapeutic treatments for motor impairments following perinatal injuries”^32^. First evidence for the responsiveness of aMF for transcranial direct current stimulation has been found in patients after stroke^33^. Also, antibodies may be used to counteract signals inhibiting axonal growth. After administering antibodies directed against the myelin protein NogoA to rodent models of stroke, enhanced sprouting of corticofugal fibers originating in the contralesional hemisphere and targeting the ipsilesional red nucleus has been observed and was associated with improved motor function^31^. Forthcoming advances in our understanding of post-lesional neuroplastic reorganization will provide means to manipulate naturally occurring processes, quite possibly aiming to optimize the collaboration of the pyramidal system and cortico-rubro- or cortico-reticulo-spinal fibers in order to amplify the endogenous potential for motor recovery.

## Methods

### Subjects

Twenty-five patients (13 women; mean age ± SD: 21.1 ± 7.6 years) who underwent a hemispherotomy using a transsylvian approach^21^ (at mean age ± SD: 12.4 ± 8.1 years) were included in this study. All patients were treated at the Department of Epileptology at the University of Bonn Medical Center and hemispherotomy was performed at the Department of Neurosurgery at the University of Bonn Medical Center between 1992 and 2012. Inclusion criteria were: (1) treatment with hemispherotomy, and (2) the ability to undergo a two-hour MRI scan, in addition to several hours of language and motor testing over two consecutive days. For analyses, patients were grouped according to their pathologies into “vascular”, “developmental”, and “progressive disorders”. Additionally, twenty-six healthy age- and gender-matched controls (16 women; mean age ± SD: 23.31 ± 7.28 years) without neurological or psychiatric diseases underwent the same scanning protocol. This study was approved by the Institutional Review Board of the medical faculty of the University of Bonn. All methods were performed in accordance with the guidelines and regulations of this ethics board and in accordance with the Declaration of Helsinki. Informed consent was obtained from all participants and/or their legal guardians.

### Functional motor assessment

The motor function of each patient’s affected upper extremity (^ue^MF) was quantified using *Fugl-Meyer Assessment*^34^. For differential analyses of proximal and distal motor skills, two new sub-scores were built. Items assessing ^prox^MF such as shoulder retraction, elevation, abduction, external and internal rotation, flexion, as well as elbow extension and flexion were grouped into one subscore (maximal value: 15). Items representing ^dist^MF, such as mass extension and flexion, flexion in proximal and distal interphalangeal joints, extension in metacarpophalangeal joints, thumb adduction and opposition as well as cylinder, spherical grip, and all subitems assessing distal motor function as part of arm movements were grouped into a *Fugl-Meyer Hand* subscore (maximal value: 6). Additionally, an index finger tapping test was performed^35^. FTR of the contralateral index finger was assessed over 20 seconds, averaged over two trials, and then used for further analysis.

### Image acquisition

Using a 3 T scanner (Magnetom Trio®, Siemens Healthineers), a 3D T1-weighted sequence (voxel size = 1.0 × 1.0 × 1.0 mm^3^, TR = 1570 ms, TE = 3.42 ms, flip angle = 15°), a 3D T2-weighted sequence (voxel size = 1.0 × 1.0 × 1.0 mm^3^, TR = 3200 ms, TE = 455 ms, flip angle = 120°), and a diffusion tensor imaging single shot, dual echo, spin echo, echo planar imaging sequence (voxel size = 1.72 × 1.72 × 1.7 mm^3^, TR = 12000 ms, TE = 100 ms, flip angle = 90°) with 60 directions and a *b*-value of 1000 s/mm² as well as six volumes with a *b*-value of 0 s/mm² were acquired for all subjects in addition to other MR sequences, which are not the focus of the current study. An eight-channel head coil was used for signal reception.

### Preprocessing

Data was preprocessed using FMRIB’s Software Library 5.0^36^ and the Tolerable Obsessive Registration and Tensor Optimization Indolent Software Ensemble (TORTOISE)^37^. For T1- and T2-weighted volumes, brain extraction was performed using FMRIB’s Brain Extraction Tool followed by bias-field correction of the data using FMRIB’s Automated Segmentation Tool^38^. T1-weighted volumes were normalized to the Montreal Neurological Institute template (MNI 152, 1 mm^3^) using a 12 degrees of freedom affine registration, performed with FMRIB’s Linear Image Registration Tool and a non-linear registration of the T1-weighted volume to MNI space, performed with FMRIB’s Non-linear Image Registration Tool^39^. In patients, lesion-masks were defined manually to exclude the lesion and only include voxels representing healthy brain tissue for the computation of the normalization warp-field.

For DTI volumes, susceptibility-induced geometric distortions were corrected by means of constrained registration^40^ together with motion and eddy current correction using TORTOISE after non-brain tissue was removed by FMRIB’s Brain Extraction Tool^41^. FMRIB’s Diffusion Toolbox^42,43^ was used to calculate the fractional anisotropy (FA) value and the probability distribution of fiber directions for each voxel. The mean of the *b*0 images was used as reference image in diffusion space and linearly registered to the respective T1 volume. If not indicated otherwise, default parameters were used.

### Tractography

Probabilistic tractography was performed for reconstruction of the PT, ^II^aMF and ^x^aMF using FMRIB’s probtrackx2^44^. Regions of interest (ROIs) for tractography were manually defined on the individual FA maps of all subjects on axial slices in native space under consistent criteria by the same operator. ROI delineation was visually guided by intensity differences on the FA map as well as by color-coded diffusion directions of the overlaid principal vector map. Three different ROIs were defined on axial slices: in the lower pons at the level of the inferior cerebellar peduncles, in the internal capsule, and in the juxtacortical white matter of the frontal lobe. For the PT, ROIs were delineated in the anterior pons (basis pontis) and for aMF, in the posterior pons (tegmentum pontis) (z in MNI space ≈ 38). The posterior limb of the internal capsule (IC) was defined as additional ROI (z in MNI space ≈ 75). A rim of white matter adjacent to the posterior bank of the precentral gyrus (M1) and extending from the deepest point of the central sulcus to the lateral crest of the precentral gyrus constituted the most superior ROI (z in MNI space ≈ 126). For the reconstruction of ^II^aMF and PT only unilateral/contralesional ROIs were applied. For the reconstruction of ^x^aMF, tractography was operated with the ipsilesional pontine and the contralesional IC and M1 ROI. Exclusion masks were defined in the corpus callosum. Additionally, pontine ROIs not belonging to the respective tracts were set as exclusion masks. Tractography was run in two directions: using the precentral gyrus ROI as a seed region, the IC ROI as a waypoint mask, the pontine ROIs as waypoint/termination mask and vice-versa. After reconstruction, tracts of both directions were added to a single tract and modified with a three percent threshold. This left us with three tracts (see Fig. 3): Unilateral/contralesional PT, unilateral/contralesional ^II^aMF, and ^x^aMF originating in the contralesional hemisphere and crossing the midline to the ipsilesional hemisphere in the pons. Finally, tracts were cropped at the level of the internal capsule to avoid the inclusion of voxels belonging to several tracts in regions where tracts overlap^7,33^. FA values of all voxels belonging to the respective tract between the internal capsule and the pontine ROIs were averaged and taken for further analysis as *tract-specific* FA values.

The canonical tracts displayed in Fig. 3 were generated by a four-step routine: (1) Voxel intensity values of non-thresholded (!) native tracts were (numerically) normalized by the number of voxels of the seed ROI, (2) tracts were (spatially) normalized to MNI-space using computed transformation matrices, (3) voxel intensity values were additionally (numerically) normalized by the highest voxel intensity value of the respective tract, and, lastly, (4) the tracts were modified using a three percent threshold and displayed in three-dimensional space (Fig. 3).

### Statistical analysis

Statistical analyses were computed with *Stata/IC 14.2 for Mac (StataCorp)*. Regression models were run to explain motor function based on patient clinical data and tract-specific FA values. Firstly, the most relevant clinical explanatory variables of ^ue^MF were identified using a stepwise regression analysis among the following possible influential factors: *time after surgery*, *age at surgery*, *gender (female | male)*, *affected hemisphere (left | right)* and *etiology (vascular | developmental | progressive disorders)*. Statistical significance was determined as *p* ≤ 0.05. Secondly, four multiple regression analyses were computed to explain ^ue^MF, ^prox^MF, ^dist^MF and FTR, respectively, based on imaging explanatory variables such as tract-specific FA of PT, ^II^aMF, and ^x^aMF. Estimators’ robustness was confirmed using bootstrapping, (10,000 replications) initiated by a reproducible randomly set seed. Multiple regressions including tract-specific FA values of more than one tract were checked for multicollinearity with variance inflation factor analysis (VIF). Group- or tract-wise FA-differences were tested using two-tailed unpaired *t-*tests.

## Data Availability

The data that support the findings of this study are available on request from the corresponding author. The data are not publicly available as they contain information that could compromise the privacy of research participants.
